# *Ulva* Seaweed-Derived Ulvan: A Promising Marine Polysaccharide as a Sustainable Resource for Biomaterial Design

**DOI:** 10.3390/md23020056

**Published:** 2025-01-24

**Authors:** Rizfi Fariz Pari, Uju Uju, Safrina Dyah Hardiningtyas, Wahyu Ramadhan, Rie Wakabayashi, Masahiro Goto, Noriho Kamiya

**Affiliations:** 1 Department of Applied Chemistry, Graduate School of Engineering, Kyushu University, 744 Motooka, Fukuoka 819-0395, Japan; pari.rizfi.784@s.kyushu-u.ac.jp (R.F.P.); rie_wakaba@mail.cstm.kyushu-u.ac.jp (R.W.); m-goto@mail.cstm.kyushu-u.ac.jp (M.G.); 2 Department of Aquatic Product Technology, Faculty of Fisheries and Marine Sciences, IPB University, Bogor 16680, Indonesia; safrina_dyah@apps.ipb.ac.id (S.D.H.); wahyu.ramadhan@apps.ipb.ac.id (W.R.); 3 Surfactant and Bioenergy Research Center (SBRC), IPB University, Bogor 16143, Indonesia; 4 Center for Coastal and Marine Resources Studies (PKSPL), International Research Institute for Maritime, Ocean, and Fisheries (i-MAR), IPB University, Bogor 16127, Indonesia; 5 Division of Biotechnology, Center for Future Chemistry, Kyushu University, 744 Motooka, Fukuoka 819-0395, Japan

**Keywords:** cellulose, green seaweed, marine biomaterial, ulvan

## Abstract

Green seaweed is currently underused compared with other major seaweed types. Many scientists have reported applications of the green seaweed *Ulva* in various fields in recent years, which makes it a candidate for biomass production in industrial biorefineries. *Ulva* contains a unique polysaccharide called ulvan, which is being considered for medicinal and pharmacological applications. Ulvan is a sulfated polysaccharide including rhamnose and glucuronic acid residues, which has a range of bioactivities, including immunomodulatory, antimicrobial, and anticoagulant properties. The biocompatibility of ulvan makes it a versatile candidate for biomaterial design. This review presents an in-depth analysis of the potential applications of ulvan, starting with extraction methods and structural/biological characterization and moving on to biomaterial design. We also highlight the advantages of ulvan over traditional seaweed polysaccharides such as agar, carrageenan, and alginate.

## 1. Introduction

Seaweed (macroalgae) has gained attention as a sustainable resource for food and non-food industries, including food additives, animal feeds, pharmaceuticals, cosmetics, textiles, bioplastics, and biofuels [1,2,3,4,5,6]. Seaweeds are classified into three groups on the basis of their pigments: red (Rhodophyta), green (Chlorophyta), and brown (Phaeophyta) [4,7]. Red seaweeds include 4000 identified species, and brown seaweeds 1500; they are more extensively studied and commercially produced than green seaweeds, of which there are 900 species [8]. These simple aquatic plants thrive in environments ranging from freshwater to highly saline oceans [7]. Seaweed is highly autotrophic, with carbon uptake rates of 8–20 tons per hectare per year [4,9,10], contributing to carbon sequestration and mitigation of climate change [11]. Its photosynthetic efficiency is 3–5 times higher than that of terrestrial plants [7,10], effectively converting sunlight and CO_2_ into biomass. For instance, brown seaweed yields up to 225 tons dry weight per hectare per year, 2.3 times higher than sugarcane [10]. Seaweeds can be harvested from the wild, and certain species are also suitable for cultivation [12]. Seaweed farming offers several advantages over terrestrial agriculture, requiring no arable land, avoiding competition with crops, and conserving resources because seaweed does not require freshwater or chemical fertilizers [13]. Additionally, its low lignin content [6,14,15,16] compared with terrestrial plants makes component extraction easier and more energy-efficient.

Green seaweed, particularly species of the genus *Ulva*, is promising because of its availability, sustainability, and diverse potential applications. Commonly known as sea lettuce, *Ulva* is found worldwide [17,18]. It demonstrates remarkable adaptability, thriving in marine, brackish, and freshwater environments [19,20]. Some *Ulva* species are associated with “green tide” blooms, which are exacerbated by seawater eutrophication and rising temperatures [21]. The largest green tide occurred in China’s Yellow Sea, covering 1746 km^2^ and producing >24 million tons of biomass [22]. *Ulva* grows in various habitats, including tidal pools and rocky shores, with optimal growth in water temperatures of 20–30 °C [17,23,24]. Its daily growth rate reaches 20.1 ± 1.8% in open seas and up to 27.9 ± 4.4% in ponds or tanks [18]. Studies indicate that *Ulva* achieves higher growth rates than other widely cultivated seaweeds, such as *Porphyra umbilicalis*, *Chondrus crispus*, and *Laminaria saccharina* [25,26].

Ulvan is a unique sulfated polysaccharide derived from *Ulva* species, known for its diverse biological activities and potential applications. It is typically extracted using direct individual extraction, which is performed separately for each product and often generates waste and environmental issues [27]. Adopting an integrated biorefinery approach [6,28,29,30,31,32,33] can enhance the production of ulvan and its byproducts, increasing the added value of the *Ulva* seaweed industry. Although several reviews have comprehensively covered ulvan [34,35], few studies have focused on biorefinery concepts using green extraction methods that highlight the potential of ulvan biomaterial.

In the context of biomaterial design, the creation and engineering of architectural biomaterials that can interact effectively with biological systems is critically important. To achieve this, the development process should encompass several critical aspects, including the selection of source materials (prioritizing sustainability), material functionalization, optimization of structural properties, and the specific design of biomaterials tailored to their intended applications. These applications may range from serving as bioactive agents to acting as the primary backbone of scaffolds. A growing trend is to use functional hybrid scaffolds made from biomaterials with bioactivity for wound healing [36] and bone tissue engineering applications [37]. Ultimately, the suitability and scalability of the source material are pressing concerns. Green seaweed, particularly *Ulva* species, has emerged as a highly promising raw material for biomaterial development because of its sustainable nature and broad applicability within the biomaterials domain.

Furthermore, the combination of natural biomaterials with advanced chemical modifications can yield materials with enhanced properties, such as improved biocompatibility, mechanical strength, and bioactivity, while maintaining environmental sustainability. Although some studies have explored ulvan, there remains a lack of comprehensive reviews focusing on its transition from resource-extraction-based research to biomaterial design applications, particularly addressing chemical and biological engineering aspects. This review aims to fill that gap by examining the comparative advantages of ulvan, its properties relative to other seaweed-derived polysaccharides, and the challenges faced in advancing ulvan biomaterial design across various fields.

## 2. *Ulva* and Ulvan—Structural Characteristics and Extraction

### 2.1. Ulva

**Figure 1:**
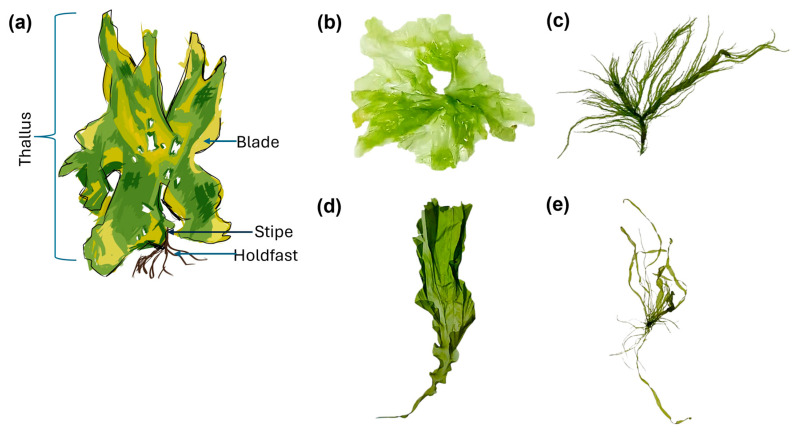
Schematic illustration of *Ulva* (**a**) and photographic representations of *Ulva* species: *U. lactuca* (**b**); *U. prolifera* (**c**); *U. linza* (**d**); and *U. flexuosa* (**e**). Reproduced from Xia et al. [40] with permission. Copyright (2023) Elsevier.

In *Ulva*, the entire body, known as the thallus, consists of a holdfast, stipe, and blade (Figure 1a), which functionally resemble the root, stem, and leaves of higher plants such as seagrass. The thallus can grow up to 30–50 cm in length [38], with its color ranging from bright to dark green, depending on the species and environmental conditions [39]. This green coloration is due to the dominance of chlorophyll pigments. The thallus is typically thin, flat, and sheet-like, resembling lettuce leaves, although some species may appear tubular or filamentous [12,40]. Examples of *Ulva* species and their shapes are shown in Figure 1.

*Ulva* contains a wide range of nutrients, including carbohydrates, proteins, fats, minerals, and vitamins. *Ulva* is composed mainly of carbohydrates (47% dw–67% dw) [41], consisting of starch (4% dw), cellulose (9% dw–10% dw), hemicellulose (14% dw–32% dw), and ulvan (13% dw–39% dw). The protein content of *Ulva* is 12% dw–30% dw and fat 1% dw–4% dw [27,28,29,41]. *Ulva* also contains macro minerals such as sodium (351–364 mg/100 g), potassium (209–467 mg/100 g), and calcium (180–1828 mg/100 g), along with trace elements such as iron (14–34 mg/100 g), zinc, copper (both 1.8 mg/100 g), selenium (1.6 mg/100 g), and manganese (4.8 mg/100 g) [42,43,44]. It also contains vitamins A (0.5 IU per 100 mg), B_1_ (1–70 ppm), B_2_ (2–40 ppm), B_3_ (2.1 ppm), B_12_, (0.64 ppm), C (2–90 ppm), and E (25.8 mg/kg dry-weight) [45,46].

### 2.2. Ulvan Chemical Structure

Water-soluble ulvan is found in the cell wall of *Ulva* together with the other polysaccharides insoluble cellulose, alkali-soluble β-1,4-D-xyloglucan, and β-1,4-D-glucuronan. Ulvan is bonded by hydrogen bonding and ionic interactions as a component of the cell wall [47]. Polysaccharide-degrading enzymes, including cellulase, pectinase, and β-glucuronidase, can degrade the cell wall of *Ulva* [48].

**Figure 2:**
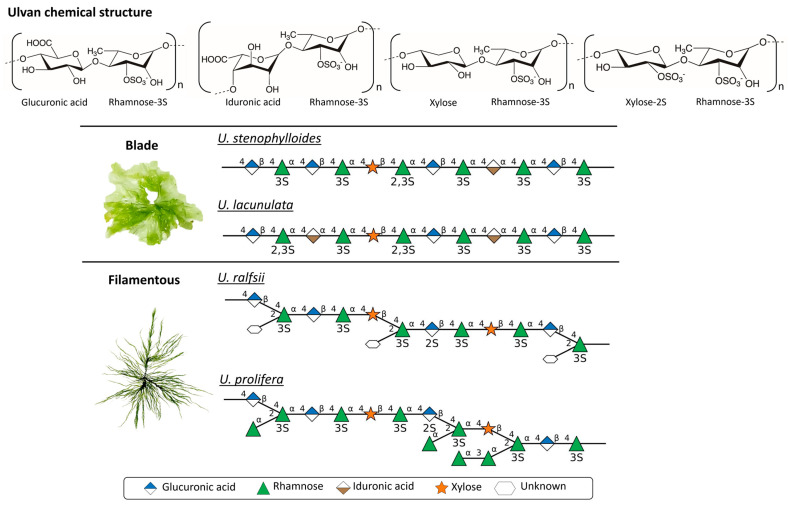
Chemical structures of ulvans. Drawings of ulvan glycan polysaccharides [50] from blade-type and filamentous-type *Ulva* species following the Symbol Nomenclature For Glycans (SNFG) provisions, produced using DrawGlycan-SNFG software (version DrawGlycan2.0) [51]. The image of filamentous *U. prolifera* is reproduced by Xia et al. [40] with permission. Copyright (2023) Elsevier.

The chemical structure of ulvan consists of repeating sugar units that are unique to individual *Ulva* species (Figure 2). The structure and functional properties of ulvan were well described by Lahaye and Robic [47]. The major monosaccharides are rhamnose (up to 92.2 mol%), glucuronic acid (up to 52.0 mol%), xylose (up to 38.0 mol%), and iduronic acid (up to 15.3 mol%) [35]. A sulfate group is attached to all rhamnose and some xylose monomers [49]. On the basis of thallus morphology, *Ulva* can be divided into blade and filamentous types. The repeating sugars in the ulvan polysaccharides of the blade and filamentous *Ulva* are different. In a recent study, it was found that sulfate is present in the glucuronic acid in ulvan from the filamentous *U. ralfsii* and *U. prolifera* [50]. Ulvan also has a carboxyl group on uronic acid in the form of glucuronic acid and iduronic acid residues. The presence of many hydroxyl groups makes ulvan hydrophilic and water-soluble.

### 2.3. Ulvan Extraction Strategies

**Figure 3:**
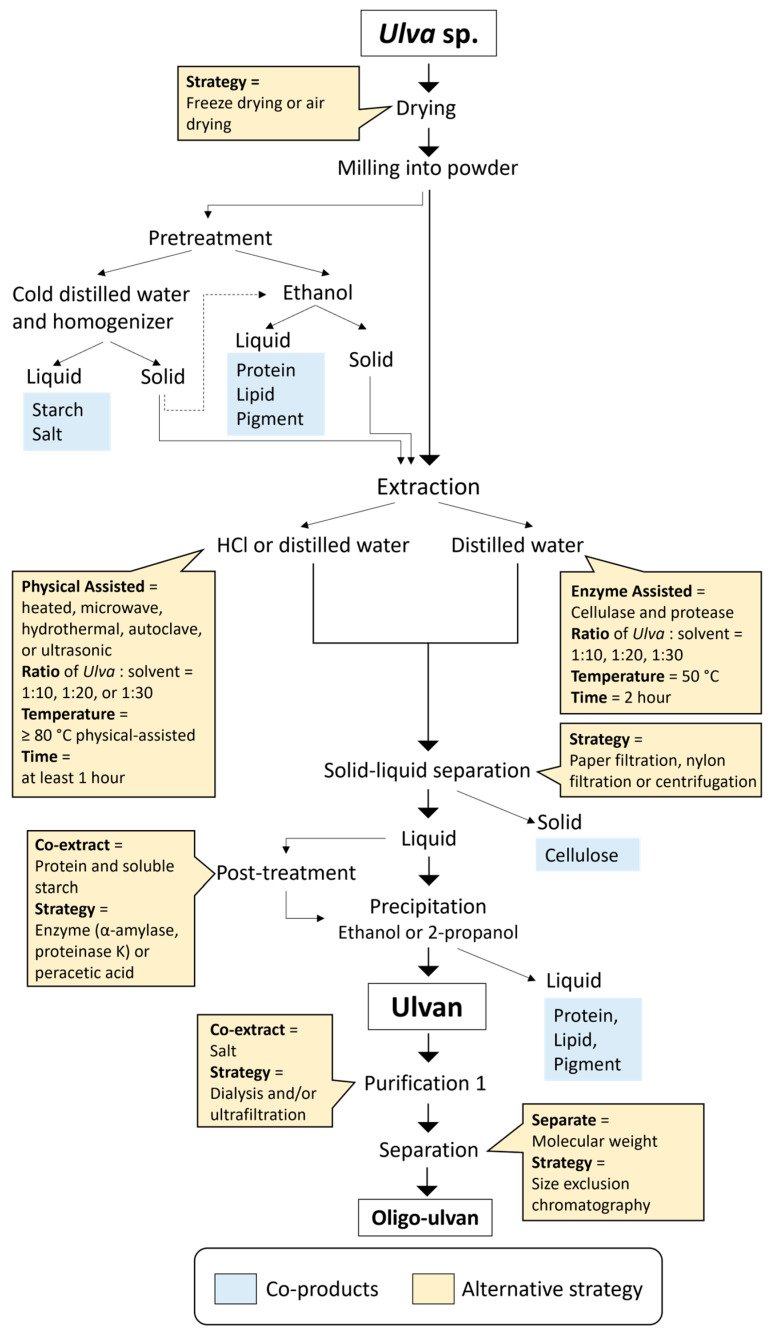
Schematic illustrating extraction steps of ulvan from *Ulva* spp. in a biorefinery concept.

In ulvan extraction, the quantity and quality of the extracted ulvan depend on the extraction method, purification process, and biomass source. The extraction method is selected based on the physicochemical characteristics of the ulvan molecule and its interactions with other components [52]. Broadly, ulvan extraction can be categorized into three approaches: physical, chemical, and enzymatic. The extraction process primarily focuses on ulvan because of its high abundance; however, co-extraction during the process often results in the presence of salts, proteins, pigments, and other polysaccharides (such as cellulose, glucuronan, xyloglucan, and starch). Figure 3 shows a schematic illustration of ulvan extraction from *Ulva*. Here, we will discuss the steps of ulvan extraction, emphasizing yield, strategies, and the management of side products in the context of *Ulva* biorefineries.

In the biorefinery concept, the goal is to optimize the use of biomass resources by maximizing benefits and profitability while minimizing waste [53]; therefore, focusing on ulvan as the primary extraction target while managing other materials through sequential extraction is a highly attractive approach. A biorefinery process can improve ulvan yield from 26% to 39% and boost cellulose recovery from 12% to 39%, compared with direct individual extraction [27]. In the context of an ulvan biorefinery, the products can be categorized into co-products, ulvan itself, and residual products.

The co-products in *Ulva* include salts, pigments, lipids, and proteins. Extracting co-products helps to decrease the co-extracted impurities in ulvan, thereby increasing its yield. Because *Ulva* is harvested from the sea, it has a high salt content, which can impact the extraction process. Decreasing the salt content enhances ulvan and protein yield while decreasing the mineral content. For instance, washing *Ulva* with distilled water at 25 °C for 30 min removes 22% of the salt without significantly affecting ulvan extraction [54]. In addition to salt, starch can be extracted using cold distilled water. Extraction of starch from *U. ohnoi* with cold distilled water and a homogenizer yields 7.33% of the dry weight of the *Ulva* [29].

Pigments are another valuable co-product, which can be extracted using ionic liquids or organic solvents [55]. Ethanol is the most commonly used solvent for pigment extraction from *Ulva*, yielding 3.6 mg/g of dry weight [56]. Cascading extraction, starting with salt removal, increased the ulvan yield from 3.7% to 8.2% of *Ulva* dry weight, while pigment extraction before ulvan extraction had no significant impact on ulvan yield [57]. In addition to pigments, ethanol also extracts lipids and proteins from *Ulva* [57,58]. The compounds extracted with an ethanol-to-water ratio of 70:30 exhibit bioactivity as an antioxidant, with an IC_50_ of 1.2 g/mL of biomass in the solvent [59]. Although pigment removal at the beginning of the process has minimal effect on ulvan yield, the pigments themselves have significant applications in fields such as cosmetics, food additives, and pharmaceuticals; therefore, their early extraction has considerable value on its own.

The extraction of ulvan requires careful optimization of solvents and conditions because these not only influence the ulvan yield but also affect the co-extraction of other materials. Table 1 summarizes the ulvan yields, molecular properties, and extraction conditions for various *Ulva* species. The solvents used for ulvan extraction are typically categorized as acidic, alkaline, or neutral. Acidic solvents generally produce higher yields compared with alkaline solvents [58]. Interestingly, even hot distilled water, a neutral solvent, can achieve good ulvan yields with a wide range of molecular weights [35]. Acidic solvents likely enhance ulvan extraction by breaking down the cell wall matrix and disrupting ionic interactions between ulvan and other components, making the polysaccharide more accessible for solubilization. In contrast, alkaline solvents may lead to degradation or selective solubilization of other polysaccharides, decreasing ulvan recovery. Another alternative is the use of deep eutectic solvents (DES), popular green solvents formed by combining two solids through hydrogen bonding. DES, such as choline chloride–glycerol, combined with post-treatment using peracetic acid, can extract ulvan from *U. lactuca* [60]. The potential of DES in ulvan extraction remains significant, but research on this method is still limited.

In addition to solvent selection, an extraction temperature of at least 80 °C is recommended for optimal results in ulvan extraction. Assisted extraction strategies, such as microwave, ultrasonic, autoclave, hydrothermal, or enzymatic techniques, can further enhance ulvan yield. Physical-assisted methods, such as microwave and ultrasonic techniques, improve extraction by disrupting cell walls through rapid heating or mechanical vibrations [58,61]; however, while physical-assisted methods are highly effective in increasing yield, they tend to consume significantly more energy compared with enzymatic approaches. Enzymatic assistance using cellulase and protease is energy-efficient and achieves higher yields at a milder temperature (50 °C) compared with non-assisted methods [62]. Enzymatic approaches target specific bonds, such as glycosidic linkages, to release ulvan with minimal structural damage and energy use [63].

The solid fraction generated during ulvan extraction constitutes a significant portion of the residual product. This solid fraction contains cellulose, protein, and minerals, with its composition varying based on the pretreatment and extraction processes used. For instance, the residual solid obtained from hydrothermal extraction combined with supercritical CO_2_ and ethanol pretreatment is rich in cellulose and protein but has low heavy metal and mineral content, making it a promising candidate for food or feed applications [64,65]. The cellulose derived from *Ulva* exhibits unique characteristics, which makes it suitable for a wide range of applications. Separating protein and cellulose from the residual solid can yield valuable side-products, enhancing the overall economic and environmental sustainability of the ulvan biorefinery process.

**Table 1:** Ulvan yield, molecular weight, sulfate content, extraction conditions, and co-products for different *Ulva* species.

Species	Yield (% dw)	MW (kDa)	Sulfate (% dw)	Extraction Solvent	T (°C)	Assisted	Solvent for Co-Product	Ref.
*U. ohnoi*	3.50	105	17.60	HCl (pH2)	37	-	-	[66]
*U. tepida*	3.90	313	21.60	HCl (pH2)		-	-	
*U. prolifera*	6.70	246	16.60	HCl (pH2)		-	-	
*U. lactuca*	17.95	-	17.22	Distilled water	50	Cellulase and protease	-	[62]
*U. lactuca*	16.90	265	53	NaOH	70	Ultrasonic		[61]
	14.50	280	58	HCl	70			
	12.50	304	39	Distilled water	70			
*U. linza*	17.00	-	-	Citric acid	60	-	-	[66]
*U. fasciata*	6.02	-	14.92	Distilled water		-	Ethanol-protein and pigment	[67]
	7.34	-	12.73	HCl		-		
	6.74	-	7.760	Na_2_EDTA		-		
*U. lactuca*	14.22	-	16.82	HCl (pH2)	80	-	-	[62]
*U. linza*	29.33	16	13.78	Oxalic acid		-	Distilled water—Starch	[66]
*U. intestinalis*	17.76	300	-	Distilled water		-	-	[58]
	23.21	88	-	Acidic water (pH3)		-	Ethanol-lipid and pigment	
	16.11	110	-	Alkaline water (pH10)		-		
	20.41	-	-	Distilled water		Microwave		
	17.89	-	-	Distilled water		Autoclave 121 °C		
	23.73	-	-	Distilled water		Ultrasonic		
*U. ohnoi*	8.20	10.5	12,5	HCl	85	-	Distilled water—salt	[57]
	7.00	16.3	12.4	HCl		-	Ethanol—pigments	
	8.10	10.8	12.5	HCl		-	Distilled water—salt Ethanol—pigments	
*Ulva* sp.	0.04	-	18.00	Citric acid	90	-	-	[68]
*U. fenestrata*, *U. lactuca*	18.00	-	17.80	HCl		-	-	[63,69]
*U. compressa*	18.00	-	17.80	HCl		-	-	[69]
*U. lactuca,*	11.00	-	14.30	Distilled water		Post-treatment α-amylase and proteinase K	Ethanol-protein and pigment	
*U. compressa*	11.00	-	9.30	Distilled water				
*U. lactuca*	41.96	-	23.20	ChCl-glycerol		Peracetic acid	-	[60]
*U. lactuca*	3.40	-	15.65	HCl (pH1.5)		-	-	[62]
*U. pertusa*	17.80	283	13.20	Distilled water		-	-	[70]
	20.60	352	9.20	Distilled water		Ultrasonic	-	
	25.30	404	6.80	HCl (pH4.5)		Pretreatment cellulase at 50 °C	-	
	26.70	300	3.90	HCl (pH4.5)			-	
*Ulva* sp.	30.36	-	-	HCl (pH2)	90	-	-	[71]
	30.48	-	31	Distilled water	120	Microwave hydrothermal	-	
	30.46	-	40	Distilled water	140		-	
	30.66	-	50	Distilled water	160		-	
	30.70	-	20	Distilled water	180		-	
	30.66	-	21	Distilled water	200		-	
*Ulva* sp.	11.00	-	11.02	Distilled water	120	Hydrothermal	Pretreatment supercritical CO_2_ and ethanol—polyunsaturated rich lipids and phenolic content	[65]
	19.00	-	7.14	Distilled water	140			
	22.00	-	10.09	Distilled water	160			
	5.00	-	7.58	Distilled water	180			
	5.00	-	7.36	Distilled water	200			

During the extraction of ulvan, various other components are co-extracted, which are typically considered impurities. To obtain pure ulvan, these impurities must be effectively removed. Ulvan, a water-soluble polysaccharide, is insoluble in alcohol [5]. This property allows for its selective precipitation while other soluble compounds remain in the liquid phase. Even with extended pretreatment strategies, the precipitation step remains critical for isolating pure ulvan [57]. This step separates ulvan from pigments, lipids, and proteins. Subsequent processes, such as centrifugation, evaporation, and ultrafiltration, are employed to separate the liquid and solid phases following precipitation.

Alcohol precipitation is a convenient and widely used method for ulvan precipitation; however, its effectiveness is limited by the insolubility of certain impurities, such as salts and starch. Post-treatment using enzymes or strong chemicals, such as peracetic acid, provides an effective means of minimizing co-extracted components [5]. For example, α-amylase can be used to decrease starch content, while proteinase K is effective for protein removal [72]. Dialysis and ultrafiltration are commonly used to remove salts from ulvan extracts. Before dialysis, the precipitated ulvan must be rehydrated using distilled water or an appropriate buffer solution. Dialysis typically employs membrane tubing with a molecular weight cut-off of 12–14 kDa [73]. The choice of downstream processing steps for ulvan purification depends on its intended applications because ulvan has a wide molecular weight range, from >400 kDa to <3.9 kDa [35].

Further purification of ulvan is performed using column chromatography, which is divided into ion exchange chromatography (IEC) and size exclusion chromatography (SEC). Because ulvan is an anionic polysaccharide, IEC using Q Sepharose XL or DEAE-Sepharose columns with 0–2 M NaCl for elution is suitable for ulvan purification [74]. SEC separates ulvan by particle size, determined by molecular hydrodynamic volume. It estimates molecular weight by comparing ulvan’s elution profile to a calibration curve of known standards, such as pullulan. High-performance SEC typically uses distilled water as the mobile phase on an HPLC system equipped with a refractive index detector [71,75].

## 3. Biological Properties of Ulvan

Ulvan has significant potential as a biomaterial, largely because of its exceptional biological properties. Ulvan can be used as a nutraceutical agent that has beneficial physiological functions, improves well-being, and reduces the risk of certain diseases, including inflammatory disorders, cancer, bacterial infections, and viral infections; it also acts as an immunomodulating and hypolipidemic agent [34]. This review highlights the biological activities of ulvan, focusing on its potential as a biomaterial with key attributes such as excellent biocompatibility, immunomodulatory effects, anticoagulant activity, and antimicrobial properties.

### 3.1. Biocompatibility

Biomaterials must be compatible with biological tissues, meaning they should not provoke harmful immune responses or toxicity when introduced into the body. This is a critical characteristic for materials used in implants, drug delivery systems, and tissue engineering. The biocompatibility of ulvan has been assessed by various methods, including cytotoxicity tests, hemolysis assays, and studies of cellular uptake and interactions.

Cytotoxicity testing has been performed both in vitro on cell cultures and in vivo using experimental animals [76,77]. The cytotoxicity of ulvan has been studied using a range of cell types, including fibroblast cells (e.g., mouse C3H [L929], 3T3) [78,79], macrophage cell lines (e.g., RAW 264.7, peritoneal, J774A.1) [80,81,82,83], gut cells (e.g., IPEC-1) [84,85,86], myoblast cells (e.g., mammalian L6 cells) [87,88,89], HaCaT keratinocytes [90], and Vero cells [91], as well as in animal models such as mice and rats [92]. The most used method for cytotoxicity testing is the MTT [3-(4,5-dimethylthiazol-2-yl)-2,5-diphenyltetrazolium bromide] assay, which measures cell viability by detecting the reduction of the yellow dye MTT into purple formazan crystals by metabolically active cells, indicating mitochondrial activity.

Ulvan has generally been shown to be non-toxic, with several studies reporting high cell viability across various cell lines exposed to ulvan extracts. Ulvan fractions have been examined from *Ulva* species, including *U. pertusa* [80,81], *U. intestinalis* [83,93], *U. armoricana* [84,85], *U. lactuca* [78,92,94], *U. clathrata* [95], *U. compressa* [96], and *U. prolifera* [82,97]. Furthermore, ulvan has been proven safe for mammalian L6 cells, showing no cytotoxic effects at concentrations under 90 mg/mL. Similarly, it did not show toxicity toward 3T3 cells at concentrations up to 10 mg/mL [90]. In human L929 cells, ulvan remained metabolically active after 72 h of exposure, with no decrease in cell viability [79].

The hemolysis assay, which measures hemoglobin release from red blood cells, is a simple screening method for the potential biocompatibility of a material. Ulvan extracted from *U. lactuca* demonstrated hemolytic activity, with a recorded hemolysis percentage of 12.38% at an ulvan concentration of 100 μg/mL [98]. The negatively charged oxygen groups on the ulvan interact electrostatically with the positively charged phosphatidylcholine lipids on the outer surface of red blood cells, contributing to the hemolytic activity of ulvan [99].

### 3.2. Immunomodulatory Effect

Immunomodulatory properties are a key biological activity and an essential parameter in biomaterial characterization, especially for applications involving interactions with the immune system, such as tissue engineering, wound healing, implants, and drug delivery. The ability of a biomaterial to modulate immune responses in a controlled manner ensures safety, biocompatibility, and effectiveness in these medical applications [85,86]. Immunomodulation refers to the alteration of the immune system’s response, either by enhancing immune responses (immunostimulation) or decreasing excessive immune activity (immunosuppression). This process involves the regulation of immune cell functions (such as macrophages, T cells, or natural killer cells), cytokine production, and overall immune system responsiveness. The primary goal of immunomodulation is to achieve a balanced immune response that supports effective defense against infections or tumors while preventing immune system overactivation (as seen in autoimmune diseases and allergies) [100].

The immunomodulatory effects of ulvan have been widely investigated in various macrophage cell types, including RAW 264.7 cells, mouse peritoneal macrophages, J77A.1 cells, and fish head kidney cells [101,102,103,104,105,106,107]. Moreover, the impact of ulvan on immune modulation has been explored in several models, including fish [108,109], porcine intestinal epithelial cells (IPEC-1 cell line), rats [77,97], mice [92,93,110,111], and chickens [112]. A range of probes are commonly used to evaluate the effects of ulvan on inflammation, including immune signaling molecules [e.g., cytokines such as tumor necrosis factor-alpha (TNF-α), interleukins (IL-1, IL-2, IL-6, IL-10, IL-12), C–X–C motif chemokine ligands (CXCL1, CXCL12, CXCL14), and C–C motif chemokine ligands], active metabolites [e.g., prostaglandin E2, nitric oxide (NO)], immunoglobulins (e.g., immunoglobulin M, intercellular adhesion molecule, and vascular cell adhesion molecule-1), enzymes (e.g., cyclooxygenase-2, inducible nitric oxide synthase-2, heme oxygenase-1, and myeloperoxidase), and transcription-related molecules [e.g., nuclear factor kappa-B (NF-κB) and mRNA] [80,81,82,93,97,109,110,113].

**Figure 4:**
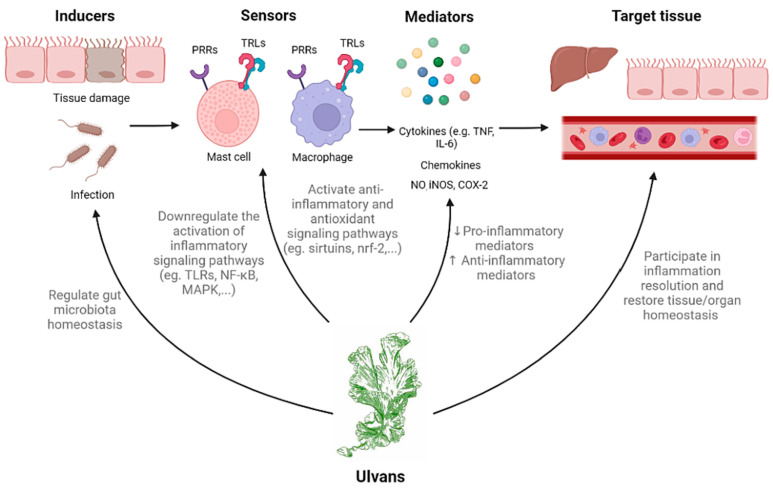
The roles of ulvans in modulating inflammatory pathways. Reproduced from Flórez-Fernández et al. [114] with permission. Copyright (2023) Elsevier.

The immunomodulatory effects of ulvans are closely associated with the inflammatory response, which can be divided into four key stages: inducers, sensors, mediators, and target tissues (Figure 4) [114]. Inflammation begins with inducers, which can be classified as either exogenous or endogenous. Exogenous inducers, such as pathogen-associated molecular patterns, are recognized by specific receptors, while endogenous inducers include damage-associated molecular patterns released from damaged host cells. Other endogenous molecules, such as advanced glycation end products and oxidized lipoproteins, are often linked to oxidative stress and can also trigger inflammation [115]. Sensors, such as toll-like receptors (TLRs), NOD-like receptors, and other pattern recognition receptors, identify these inducers and activate downstream signaling pathways such as mitogen-activated protein kinase (MAPK), NF-κB, and SIRT1/FOXO1 [116]. These signaling pathways lead to the production of various inflammatory mediators, including chemokines, cytokines, vasoactive amines, eicosanoids, matrix metalloproteinases, NO, and free radicals. These mediators are involved in several processes, such as pain induction, immune modulation, and tissue repair [115,116]. The target tissues affected by these mediators show responses such as increased vascular permeability, immune cell recruitment, and either tissue damage or repair. If this process is dysregulated, it may lead to chronic inflammation and associated disorders [80,114].

Ulvans function as immunomodulatory agents through various mechanisms that influence the immune system, including the suppression of proinflammatory cytokine production, modulation of the NF-κB pathway, enhancement of anti-inflammatory cytokines, regulation of TLR signaling, and restoration of the balance of gut microbiota (Figure 4). The immunostimulatory properties of ulvan are largely determined by its structural features, such as the monosaccharide composition, sulfate content, and molecular weight, as discussed by Kidgell [35]. One of the key mechanisms of ulvans is the regulation of cytokine production, particularly proinflammatory cytokines that are crucial in immune responses. Cytokines such as TNF-α, IL-6, and IL-1β, which are commonly elevated in inflammatory conditions such as inflammatory bowel diseases [117], can be modulated by ulvans. Additionally, ulvans regulate intestinal inflammation by inhibiting the NF-κB pathway. Ali et al. (2016) [118] demonstrated that ulvan from *U. pertusa* COMP decreases the production of proinflammatory cytokines (IL-12, p40, IL-6, TNF-α) by preventing the phosphorylation of IκBα, which inhibits its degradation and the nuclear translocation of NF-κB. This action suppresses the expression of inflammation-related genes and helps maintain immune homeostasis.

Ulvan enhances the production of anti-inflammatory cytokines, such as IL-10. This cytokine is vital for suppressing inflammatory responses and promoting tissue repair. By increasing IL-10 levels, ulvans counteract the effects of proinflammatory cytokines, further contributing to an anti-inflammatory state [119,120].

### 3.3. Anticoagulation Activity

Anticoagulant activity is a vital characteristic of biomaterials used in medical applications, especially those that come into direct contact with blood. This property ensures biocompatibility, functionality, and safety by preventing blood clot formation (thrombosis), minimizing inflammation, and maintaining proper blood flow, which collectively improve patient outcomes and extend the lifespan of medical devices [121]. The anticoagulant potential of a molecule, including ulvan, is assessed via parameters such as the activated partial thromboplastin time (aPTT), thrombin time (TT), and prothrombin time (PT), which are used to evaluate the effect on different pathways of the coagulation cascade, including antithrombin-dependent (anti-Xa and anti-IIa), intrinsic/common (aPTT), extrinsic (PT), and common (TT) pathways. The intrinsic pathway is triggered by the interaction of Factor XII with an anionic surface, while the extrinsic pathway is initiated when Factor VII binds to tissue factor, a receptor released from damaged cells. Both pathways converge at Factor X, activating the common pathway, which leads to the conversion of prothrombin into thrombin. Thrombin then catalyzes the transformation of soluble fibrinogen into insoluble fibrin, forming the structural framework of a clot [122].

Several studies have shown that ulvan from various species of *Ulva*, including *U. lactuca* [123,124], *U. prolifera* [125], *U. fasciata* [124,126], *U. nematoidea* [127], *U. conglobata* [128], *U. linza* [129,130], and *U. reticulata* [124], exhibits anticoagulant activity through the intrinsic and/or common pathways of the coagulation cascade. Ulvan from *U. nematoidea* demonstrated a high aPTT index of 1.8, indicating its effectiveness in prolonging clotting time and suggesting a strong anticoagulant effect [127]. The ulvan isolated from *U. conglobata* showed significant anticoagulant activity, primarily through direct inhibition of thrombin in a dose-dependent manner; its activity was stronger than that of heparin in the presence of heparin cofactor II [128]. The anticoagulant potency of ulvan differs between *Ulva* species and is influenced by environmental and physiological factors. The anticoagulant activity of ulvan depends on the degree of sulfation and its molecular weight [127,128,129,130]; higher sulfation enhances its activity, while molecular weights <200 kDa can diminish it [127].

### 3.4. Antimicrobial Activity

Antimicrobial activity is a crucial characteristic of biomaterials, particularly those used in medical applications because it significantly enhances their safety and functionality. It helps prevent infections by decreasing microbial colonization on the material’s surface, which is particularly important for implants, wound dressings, and other medical devices. Additionally, antimicrobial properties protect biomaterials from degradation caused by microbial activity, thereby prolonging their lifespan and maintaining their performance [131].

Ulvan has been studied for its ability to combat various harmful microorganisms. Ulvan derived from *U. reticulata* demonstrated antimicrobial properties, showing significant inhibitory effects on two pathogenic bacteria linked to skin diseases and inflammation [132]; inhibition zones of 20 mm were reported against Enterobacter cloacae and 18 mm against *Escherichia coli*. The activity of ulvan extracts from *Ulva* species against *Staphylococcus aureus*, *S. epidermidis*, and *Cutibacterium acnes* has been reported [133]. Ibrahim et al. [134] emphasized the potent antimicrobial effects of partially purified ulvan from *U. lactuca* against certain fish and human pathogens, including *E. coli* ATCC 8739, *Bacillus subtilis* ATCC 6633, *S. epidermidis*, *Aeromonas hydrophila*, *Pseudomonas fluorescens* ATCC 17386, *P. aeruginosa* ATCC 9027, *Klebsiella pneumoniae* ATCC 13883, and *Candida albicans* ATCC 10231. In addition, ulvan also demonstrated antifouling effects on glass slides towards seawater fouling bacteria, likely because of electrostatic repulsion between the negative charges on bacteria and the negatively charged carboxyl groups of ulvan [134]. This interaction significantly decreased bacterial adherence and may contribute to bacterial cell death (i.e., a bactericidal effect).

The antimicrobial mechanisms of marine polysaccharides are thought to involve: (i) disrupting the cell wall membrane of microbes, causing cytoplasmic leakage and leading to cell death, (ii) binding to glycoreceptors on the microbial cell wall, and (iii) altering microbial DNA. The antifungal effects of algal polysaccharides are believed to result from their interaction with glycoreceptors, membrane components, and nucleic acids in bacterial cells [135]. In addition, polysaccharides may not directly kill microbes but may work by sequestering nutrients, thus decreasing their availability. The antimicrobial effects of ulvan are attributable to its distinctive branched structure [132].

## 4. Ulvan in Biomaterial Design

Because of its unique structural and functional properties, ulvan exhibits significant potential in multiple fields. The unique structure comes from its β-(1,4) backbone composed of alternating glucuronic acid and rhamnose units, which are often modified by varying patterns of sulfation (Figure 2). These sulfate groups are crucial because they enable intermolecular interactions, such as hydrogen bonding and electrostatic forces, which are essential for gelation [136]. Additionally, ulvan contains carboxyl groups that serve as sites for synthesizing complexes with other molecules, further expanding its functional versatility. The natural origin of ulvan as a renewable and sustainable material enhances its relevance in environmentally friendly biomaterial design.

**Figure 5:**
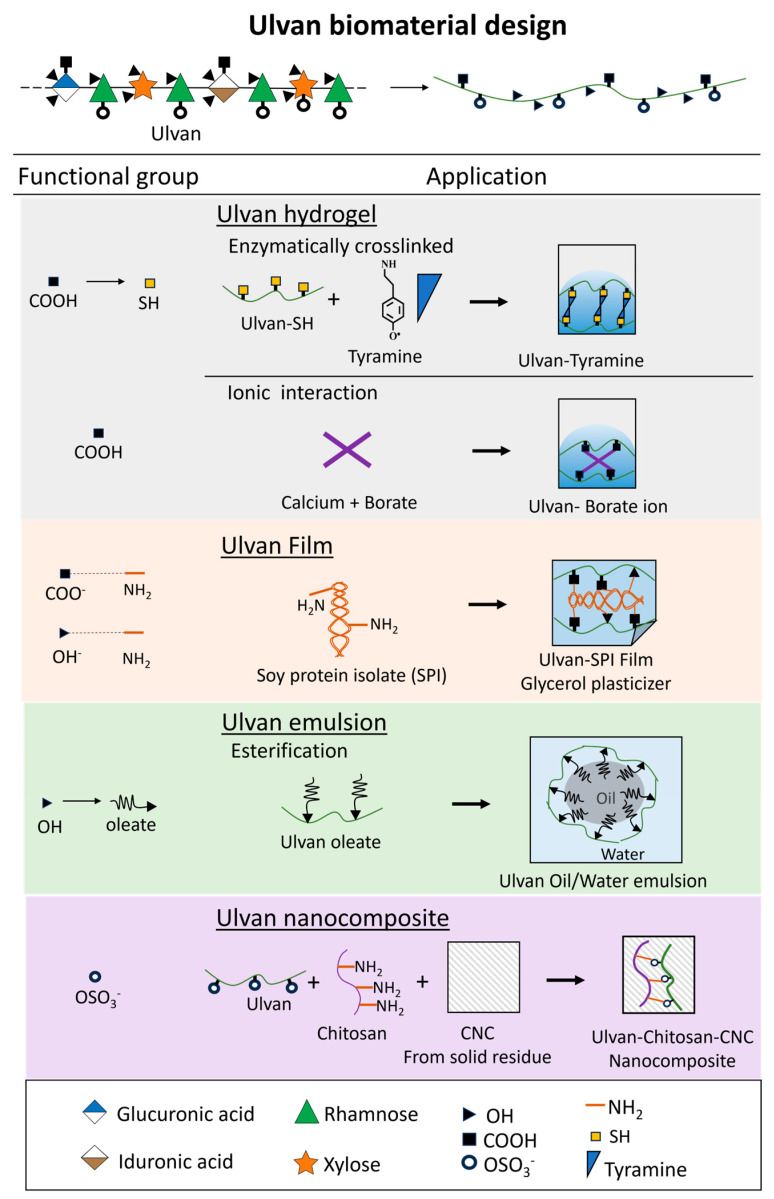
Illustration of ulvan biomaterial design strategies for hydrogels, films, emulsions, and nanocomposites.

Ulvan has the remarkable ability to transform liquids into semi-solid gels by forming network structures. This process occurs through hydrogen bonding, ionic interactions, or hydrophobic interactions. These properties make ulvan highly valuable in the food, cosmetics, and pharmaceutical industries, where it is used to improve the texture, stability, and consistency of products. We will discuss the use of ulvan in hydrogels, films, emulsions, and nanocomposites (Figure 5).

### 4.1. Ulvan-Based Hydrogels

The carboxyl and sulfate groups of ulvan play a pivotal role in its ability to form hydrogels through various crosslinking methods. One such method involves oxidizing ulvan with sodium periodate, which introduces aldehyde groups along the polysaccharide backbone [137]. A Schiff base reaction occurs when the aldehyde groups (−CHO) in ulvan dialdehyde interact with the primary amine groups (−NH_2_) in the lysine residues of gelatine, resulting in stable imine (C=N) bonds [138]. Using phosphate-buffered saline, hydrogels made from 80% gelatine and 20% ulvan showed 300% gel swelling capacity, while hydrogels made from 40% gelatine and 60% ulvan reached 900% gel swelling capacity. When deionized water was used, swelling capacity increased significantly, with 80% gelatine and 20% ulvan samples showing 1000% swelling and 40% gelatine and 60% ulvan samples exhibiting an extraordinary swelling capacity of 2400% [69]. These results demonstrate the tunable properties of ulvan-based hydrogels depending on the solvent composition and polymer ratio. The intermolecular interaction not only creates a robust hydrogel network but also enhances the binding capacity for dyes and heavy metal ions [69]. The Schiff-base strategy involving gelatin matrices is applicable to tissue engineering applications [139] and bone tissue engineering [140].

Another advanced approach to hydrogel biomaterial design in mild conditions uses enzymatic crosslinking of ulvan using horseradish peroxidase (HRP) and hydroxyphenyl compounds such as tyramine. Hydroxyphenyl compounds are particularly suitable substrates for HRP because of their reactivity in green chemistry processes [141]. Tyramine is the most commonly used hydroxyphenyl compound in enzymatic gelling systems, offering a safe profile and efficient conjugation via its terminal amine group [142]. In this system, ulvan–tetrahydroxyphenyl conjugates are synthesized by forming amide bonds between the carboxyl groups of ulvan and the amine group of tyramine, facilitated by carbodiimide chemistry [143]. The carboxyl groups are first activated by reacting with EDC and sulfo-NHS in a slightly acidic environment, creating reactive sulfo-NHS esters optimized for polymer bonding. In the presence of hydrogen peroxide (H_2_O_2_), HRP catalyzes the oxidative coupling of hydroxyphenyl compounds through a radical mechanism [142]. Covalent bonds form between carbon atoms in the ortho position to the hydroxyl or oxygen atoms of phenol groups, creating a stable crosslinked network. These enzymatically crosslinked hydrogels achieve swelling degrees of approximately 2000%, classifying them as superabsorbent materials [143]. The enzymatic and H_2_O_2_ conditions can be adjusted to optimize gelation times for applications such as injectable hydrogels [144]. Hydrogelation of ulvan with boric acid and Ca^2+^ form ionic crosslinking with weak gel properties [145].

The gelling potential of ulvan is also being explored in the development of bioinks for 3D printing applications. A selected coagulation of ulvan derived by bio ionic liquid is formulated by mixing it with choline chloride and subjecting it to ethanol precipitation. This process results in a material with excellent viscoelastic strength [71], making it an ideal candidate for use in advanced 3D bioink formulations. Another formulation of ulvan bioink, combined with gelatin methacryloyl and gelatin type A, demonstrates tunable properties, enhanced mechanical strength, self-recovery capabilities, and support for cell proliferation. Its tunable shrinkage and degradation characteristics contribute to significant improvements in scaffold resistance and wound healing [146]. More details on its biocompatibility and specific cell interaction performance would enable assessment of its relevance in biomedical applications.

The exploration of ulvan hydrogels extends to food applications. When formulated with alginate, ulvan forms a scaffold that is suitable for probiotic encapsulation. While alginate is widely used as a scaffold for nutrient encapsulation [147], it has limitations when applied to systems involving living organisms. In contrast, ulvan demonstrates strong prebiotic activity and supports cell proliferation [148], effectively overcoming the limitations of alginate in microbial encapsulation. Ulvan–alginate hydrogel beads for probiotic immobilization have been shown to enhance the survival rate of probiotics in both simulated gastric and intestinal fluids [149]. This system offers significant potential for applications in the food and beverage industry.

### 4.2. Ulvan-Based Films

Ulvan can be formulated into hydrophilic and hydrophobic films. Ulvan-based film formulations use glycerol as a plasticizer to create hydrophilic films. These films are typically composed of ulvan, glycerol, distilled water, and boric acid, enabling for wound dressing application [150]. The high solubility of ulvan hydrophilic films can also be considered as edible coating application [151]. To enhance the characteristics of the material, 10% carnauba wax and Tween-80 can be added, resulting in hydrophobic films ideal for food packaging [152]. Ulvan hydrophobic films in combination with soy protein isolate (SPI) demonstrate impressive hydrophobicity, flexibility and mechanical properties, with an average tensile strength of 3.5 MPa and an average elongation at break of 19.9% [153]. The crosslinking occurs between amine group in SPI and hydroxyl group in ulvan. Further comparisons with conventional gelling agents would provide insights into the performance and sustainability of ulvan.

### 4.3. Ulvan-Based Nanocomposites

Hybrid hydrogels that incorporate ulvan exhibit enhanced mechanical and functional properties. For instance, ulvan crosslinked with citric acid and hybridized with sodium carboxymethyl cellulose (Na-CMC) achieves a swelling degree of up to 387.5% [68], highlighting its improved mechanical stability. This hybrid hydrogel is synthesized using a single-pot heating method, where a fixed citric acid concentration of 20 wt% is used to crosslink ulvan and Na-CMC. The simplicity of this process enhances its applicability in scalable production methods. Comparatively, ulvan hydrogels without Na-CMC show a swelling capacity of 248.7%, demonstrating that hybridization significantly improves performance [68].

Comprehensive use of *Ulva* was further demonstrated by Mariia et al. [154]. Ulvan was reacted with chitosan to form polyelectrolyte complexes involving anionic and cationic side-chain interactions. The sulfate groups in ulvan carry a negative charge, while the amine groups in chitosan are positively charged. Combining chitosan/ulvan with cellulose nanocrystals (CNCs) extracted from the solid residue of ulvan extraction results in nanobiocomposite hydrogels designed for wound healing applications. The addition of ulvan in chitosan hydrogels incorporating 20% CNC shows an enhanced tensile strength of 1.2 MPa and a significant increase in swelling capacity, indicating improved mechanical properties. Furthermore, these nanocomposites exhibit excellent cell proliferation, remarkable biocompatibility, and nontoxicity. The sustained release of epidermal growth factors from these hydrogels accelerates wound healing, demonstrating their advanced therapeutic potential.

Further advancements in ulvan research include ulvan hybrids with carrageenan and polyvinyl alcohol (PVA). For ulvan hybrid carrageenan, the synthesis of ulvan–amide derivatives proceeds through carbodiimide chemistry. These derivatives enable the creation of ulvan–k-carrabiose hybrid polysaccharides, expanding the scope of ulvan-based materials [155]. For ulvan nanofiber fabrication with PVA, ulvan–PVA nanofibers, produced via electrospinning with a 1:2 ulvan/PVA ratio, demonstrate promise for advanced material applications, including biomedical and environmental uses [156]. Specific examples of these applications are filtration membranes or antimicrobial coatings.

### 4.4. Ulvan-Based Emulsions

A detailed evaluation of ulvan highlights its significant potential in various industries, especially for food and cosmetic formulations. The capacity of ulvan to hold water and oil, quantified at 3.17 g water/g ulvan and 2.66 g oil/g ulvan [58], makes it particularly useful for stabilizing emulsified products. This capability helps prevent liquid separation (syneresis) while enhancing viscosity and texture, ensuring consistency in complex formulations [157].

In emulsified systems, increasing ulvan concentrations from 1% to 3% enhances both emulsion activity and stability. At the peak concentration of 3%, ulvan achieves an emulsion activity index of 69.66 m^2^/g and a stability of 72.39% [58]. While polysaccharides are typically recognized for their stabilizing rather than emulsifying roles, ulvan stands out because of its surface-active functional groups (carboxyl, sulfate, and hydroxyl), as well as hydrophobic protein-like elements [158]. The molecular structure of ulvan, which includes a high molecular weight and glucuronic acid content, significantly bolsters its ability to form stable emulsions by increasing solution viscosity and strengthening interfacial layers [159]. To work as emulsifier agent, the hydrophilic/hydrophobic balance of ulvan could be optimized by modifying ulvan with long fatty acid like oleic acid [58,143]. The modification shows valuable functional and bioactive for flavoring agent in soft drink beverages, and stabilizing agent in body cream in cosmetic application.

Ulvan also exhibits excellent foaming capabilities, achieving a foaming capacity of 75% and stability of 54% at 3% concentration [58]. These properties are crucial for developing consistent and appealing textures in food and cosmetic products [158]. As ulvan concentrations increase, its ability to stabilize foam improves, largely because of its high molecular weight. This allows it to create robust networks at the air–water interface, effectively delaying the merging of gas bubbles and prolonging foam integrity. Such characteristics are highly beneficial for applications in whipped desserts, aerated beverages, and cosmetic mousses, where stable foam structures are essential [157,158].

## 5. Comparative Advantages of Ulvan

One of the key advantages of ulvan is its potentially high yield resulting from the high biomass of *Ulva* species, which can outperform other seaweed-derived resources such as agar, carrageenan, alginate, laminaran, fucoidan, porphyran, floridean, and xylofucoglycan [160]. Ulvan extracted from *Ulva* is derived from an undesired seaweed. *Ulva* can be produced 7–10 times faster and in higher quantities than other commercially available seaweed biomass. This characteristic ensures a sustainable production pipeline, addressing scalability issues that often limit other polysaccharides.

The extraction of ulvan is also relatively easy and simple, making it a more accessible and cost-effective option than other marine polysaccharides. Compared with other marine polysaccharides, ulvan demonstrates superior yield potential, with up to ~41% extraction efficiency from dry *Ulva* biomass [60,161]; this is higher than the yields typically reported for agar (10–15%) or fucoidan (5–10%). The ability of *Ulva* species to proliferate in eutrophic environments further enhances their suitability for large-scale production, reducing reliance on the pristine ecosystems that are required for agarophytes or carrageenophytes. Chemically, the unique properties of ulvan offer distinct advantages in functionality and bioactivity.

Notably, dissimilar to agar or carrageenan, which require heat for gel formation, ulvan can form gels in non-heated conditions through ionic cross-linking with CaCl_2_ and H_3_BO_3_ [47]. This characteristic could position it as a potential alternative to alginate, a widely used marine polysaccharide. This property is advantageous for applications requiring cold processing, such as in the pharmaceutical and food industries. For instance, ulvan-based hydrogels can encapsulate heat-sensitive bioactive molecules, which may not be the case for thermally reliant gelling agents. Furthermore, the high sulfate content of ulvan has garnered interest in biomedicine because it may confer anticoagulant properties, similar to heparin, leading to ulvan being referred to as “vegan heparin” [162]. Compared with other seaweed polysaccharides, ulvan has also demonstrated versatility in its bioactivity, exhibiting a range of beneficial properties, such as antioxidant, immunomodulatory, anticancer, and antimicrobial activities [160,163]. The richness of sulfated moieties in the structure of ulvan favors its potential for engineering and modification, opening up opportunities for the development of innovative polymeric materials and applications [164]. A comprehensive comparison between ulvan and other marine polysaccharides is shown in Table 2.

However, despite the potential benefits of ulvan discussed above, its development faces certain challenges that could hinder its broader adoption for commercial and industrial needs. 

The extraction process for ulvan remains less standardized than that for traditional polysaccharides such as agar or carrageenan. Variability in the chemical composition of *Ulva* biomass due to environmental factors affects the consistency and quality of the product ulvan. Green solvent and enzyme-assisted methods show promise for improving extraction efficiency but require further optimization for industrial scalability. Specialized extraction methods, including green solvents and enzymatic approaches, increase production costs. The absence of well-established infrastructure for ulvan processing further compounds this issue, making it less competitive than agar or alginate in certain markets.

Currently, most *Ulva* cultivation occurs in wild or semi-wild conditions; environmental factors such as temperature, nutrient availability, and seasonal variations significantly affect the yield and quality of ulvan. This lack of controlled cultivation introduces variability and limits the scalability of ulvan production. Although ulvan exhibits excellent gelling and bioactivity properties, its mechanical strength in hydrogels is often lower than that of alginate or carrageenan. This limitation restricts its use in applications demanding high structural integrity, such as wound dressings or load-bearing scaffolds in tissue engineering. To overcome these limitations, future research efforts should focus on:

Enhanced extraction techniques: Development of cost-effective, standardized methods for high-purity ulvan production.

Cross-linking strategies: Enhancement of the mechanical properties of ulvan-based materials through innovative cross-linking and composite formation.

Sustainability metrics: Incorporation of life cycle assessments to establish ulvan as a green alternative to synthetic and traditional polysaccharides.

Controlled cultivation: Advancement of aquaculture techniques for *Ulva* species to ensure stable and high-quality ulvan yields independent of seasonal and environmental variations.

Establishment of high-quality ulvan standards depending on its intended use, i.e., as feed, a food source, or a bioactive compound for application in biomedicine.

If these challenges are addressed, ulvan may become established as a versatile cornerstone material in biomaterial science and industrial applications.

**Figure 6:**
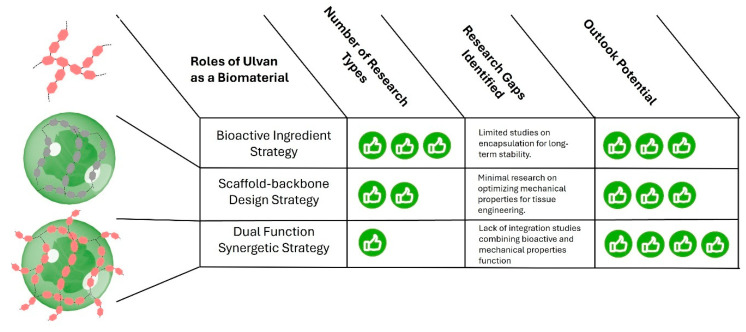
Summary of the roles of ulvan as a biomaterial and the outlook for biomaterial design.

Last, the evaluation of ulvan as a biomaterial emphasizes its potential in three key roles: as a bioactive ingredient, in scaffold-backbone design, and its combination as dual-function synergy (Figure 6). While substantial progress has been made, critical gaps remain in integrated functionality, mechanical property optimization, and encapsulation stability. Addressing these issues could unlock transformative applications for ulvan in biomaterial design, particularly in biomedicine and industry.

## 6. Conclusions

*Ulva* is a potent and versatile seaweed that is robust and sustainable. The cultivation of *Ulva* offers environmental benefits, including high carbon sequestration, rapid growth, and low resource requirements. Integrated green biorefinery approaches to *Ulva* enhance the extraction efficiency of its main polysaccharide, ulvan. This approach also enables the recovery of valuable co-products such as pigments, proteins, and cellulose, boosting sustainability. The superior yield and scalability of ulvan compared with other marine polysaccharides position it as an economically viable alternative to their use.

Ulvan, a sulfated polysaccharide, is a promising biomaterial because of its unique structure and exceptional bioactive properties, including immunomodulation, anticoagulant activity, antimicrobial effects, and biocompatibility. Its ability to form hydrogels, films, scaffolds, and nanocomposites makes it valuable for biomedical, food, and environmental applications. Dissimilar to traditional polysaccharides, the non-heated gelation of ulvan and its high sulfate content provide advantages for preserving heat-sensitive bioactive materials and mimicking heparin-like anticoagulant properties.

Despite its potential, challenges remain in the optimization of ulvan extraction and impurity management. Advancing green extraction methods and exploring innovative applications will unlock the full potential of ulvan. Based on its sustainability, versatility, and scalability, ulvan is poised to play a key role in advancing biomaterial design and addressing global challenges in healthcare, food security, and environmental sustainability.

## Figures and Tables

**Table 2:** Comparison of chemical modifications, gelling mechanisms, yield, and applications in biomaterial design of marine polysaccharides.

Polysaccharide	Source Algae	Yield Range (%)	Gelling Mechanism	Existing Function in the Current Market	Commercial Availability	Mechanical Properties	Cytocompatibility	Thermal Stability	Cross-Linking Potential	Functionalization Potential	Common Modifications	Applications of Modifications	Challenges in Modifications
Ulvan	*Ulva* spp.	15–41	Ionic cross-linking with divalent cations (e.g., Ca^2+^; CaCl_2_, H_3_BO_3_)	Emerging in tissue engineering, drug delivery, and bioadhesive development	Moderate, requires specialized extraction processes	Moderate elasticity, suitable for hydrogels	Excellent, supports cell adhesion and proliferation	Moderate, stable up to ~80 °C under physiological conditions	High, form strong ionic cross-links	High, easily modified with bioactive groups	Thiolated ulvan, sulfation, carboxymethylation, phosphorylation, hydrogel formation	Drug delivery, tissue engineering, bioadhesive development, antioxidant systems	Complexity in achieving uniform thiolation, scalability issues
Agar	*Gracilaria* sp., *Gelidiella* sp., *Gelidium* sp., *Pterocladia*, *Laurencia*	10–15	Thermal gelation via hydrogen bonding	Widely used in the food industry (gels, thickeners), limited biomedical applications	High, widely available, and established supply chain	Strong, brittle gels, limited elasticity	Good, limited applications in biomedical fields	High, retains gel properties up to ~100 °C	Moderate, limited chemical reactivity	Moderate, limited functionalization pathways	Thiolated agar, esterification, hydrogel formation, nanoparticle stabilization	Encapsulation, tissue scaffolding, bioadhesives, wound dressings	Low reactivity under mild conditions, batch variability
Carrageenan	*Kappa-phycus* spp.	20–30	Thermal gelation via sulfate groups. Helical structures formed via 3,6-anhydrous-galactose units and ion interactions	Predominantly in food as stabilizers and thickeners, some drug delivery systems	High, commercially available for various industries	Flexible gels, moderate strength	Moderate, may require modifications for biocompatibility	High, stable up to ~120 °C	High, versatile cross-linking potential	High, supports diverse chemical modifications	Sulfation, hydrogel formation, derivatization for drug delivery	Drug release matrices, bioadhesive, biocompatible scaffolds	Control over sulfation levels, stability in physiological conditions
Alginate	*Macrocystis* spp., *Laminaria hyperborea*, *Laminaria digitata*, *Laminaria japonica*, *Sargassum* sp., *Ascophyllum nodosum*	15–35	Ionic cross-linking with Ca^2+^ or other divalent ions	Extensively in food, pharmaceuticals, and wound care products	High, extensively used, and widely produced	High elasticity, robust structural integrity	Excellent, widely used in tissue engineering	High, stable across wide temperature ranges (~150 °C)	High, readily cross-links with divalent ions	High, extensively modified for various uses	Calcium cross-linking, thiolation, carboxylation, hydrogel formation, esterification	Controlled release systems, wound care, tissue scaffolding	High dependency on cross-linking agents, cost of modification processes
Fucoidan	*Fucus vesiculosus*, *Cladosiphon okamuranus*, *Laminaria japonica*, *Undaria pinnatifida*	5–10	Not a primary gelling agent, it interacts through sulfated domains	Limited use in niche biomedical applications (anticoagulants, drug carriers)	Low, niche market with limited availability	Weak mechanical properties, limited application	Variable, dependent on sulfation level	Moderate, sensitive to heat above ~70 °C	Low, limited cross-linking capability	Moderate functionalization depends on sulfate groups	Sulfation, desulfation, acetylation, hydrogel formation, anti-coagulant enhancement	Anti-inflammatory agents, drug carriers, heparin substitutes	Variability in biological activity, cost-intensive extraction and modification
Laminaran	*Laminaria* spp.	10–20	Weak hydrogen bonding and limited gel formation	Occasionally, in nutraceuticals and research-grade biomaterials	Low, specialized production with limited supply	Low mechanical strength, not a primary gelling agent	Moderate, limited data on cytocompatibility	Low, weak stability under heat, <60 °C	Low, rarely used for cross-linking	Low, not typically functionalized extensively	Oxidation, acetylation, hydrogel formation, nanoparticle delivery systems	Nanoparticle stabilizers, immune enhancement, tissue scaffolds	Low stability under physiological conditions, complex modification processes
Porphyran	*Porphyra* spp.	10–15	Thermal gelation and hydrogen bonding	Emerging in antioxidant-rich supplements and basic drug delivery systems	Moderate, emerging commercial interest	Moderate strength, suitable for soft applications	Good, supports basic biomedical applications	Moderate, stable under mild thermal conditions (~80 °C)	Moderate potential for chemical derivatization	Moderate, supports basic functionalization	Sulfation, esterification, hydrogel formation, antioxidant enhancement	Antioxidant applications, drug delivery, immune modulation	Limited structural studies, stability in industrial applications

## Data Availability

Not applicable.

## Abbreviations

The following abbreviations are used in this manuscript:

dw	dry weight
SNFG	Symbol Nomenclature for Glycans
ChCl	Choline Chloride
DES	Deep Eutectic Solvent
COMP	3-hydroxy-4,7-megastigmadien-9-one
EDC	1-ethyl-3-(3-dimethylaminopropyl)carbodiimide
NHS	N-hydroxysuccinimide
DGR	Daily growth rate
MWCO	Molecular weight cut-off
SEC	Size Exclusion Chromatography
IPEC-1	porcine intestinal epithelial cells
TNF-α	tumor necrosis factor-alpha
IL	Interleukin
CXCL	C-X-C motif chemokine ligand
IgM	Immunoglobulin M
COX-2	Cyclooxygenase-2
iNOS-2	inducible nitric oxide synthase-2
NF-κB	nuclear factor kappa-B
MAPK	Mitogen-activated protein kinase
TLR	Toll-like receptors
HRP	Horseradish peroxidase
Na-CMC	Sodium carboxymethyl cellulose
CNC	Cellulose nanocrystals
PVA	Polyvinyl alcohol
